# Label-Free Enrichment of Adrenal Cortical Progenitor Cells Using Inertial Microfluidics

**DOI:** 10.1371/journal.pone.0046550

**Published:** 2012-10-04

**Authors:** Soojung Claire Hur, Tatiana Z. Brinckerhoff, Christopher M. Walthers, James C. Y. Dunn, Dino Di Carlo

**Affiliations:** 1 Rowland Institute at Harvard, Harvard University, Cambridge, Massachusetts, United States of America; 2 Division of Pediatric Surgery, University of California Los Angeles, Los Angeles, California, United States of America; 3 Department of Bioengineering, University of California Los Angeles, Los Angeles, California, United States of America; 4 California NanoSystems Institute, Los Angeles, California, United States of America; 5 Jonsson Comprehensive Cancer Center, Los Angeles, California, United States of America; Universität Heidelberg, Germany

## Abstract

Passive and label-free isolation of viable target cells based on intrinsic biophysical cellular properties would allow for cost savings in applications where molecular biomarkers are known as well as potentially enable the separation of cells with little-to-no known molecular biomarkers. We have demonstrated the purification of adrenal cortical progenitor cells from digestions of murine adrenal glands utilizing hydrodynamic inertial lift forces that single cells and multicellular clusters differentially experience as they flow through a microchannel. Fluorescence staining, along with gene expression measurements, confirmed that populations of cells collected in different outlets were distinct from one another. Furthermore, primary murine cells processed through the device remained highly viable and could be cultured for 10 days *in vitro*. The proposed target cell isolation technique can provide a practical means to collect significant quantities of viable intact cells required to translate stem cell biology to regenerative medicine in a simple label-free manner.

## Introduction

There exists extensive interest in the enrichment and purification of specific subpopulations of cells from heterogeneous biological samples for immunology research, tissue engineering, and medicine [1]. In particular, the ability to collect viable cells of interest for downstream use may have beneficial impacts on cancer diagnosis and prognosis [2], [3], cell-based therapeutics and regenerative medicine [4]–[6], and non-invasive prenatal diagnostics [7]. Conventional technologies available for the isolation and detection of cells, such as immunolabeling-based flow cytometry and magnetic-activated cell sorting, often require well-defined molecular biomarkers, laborious immunolabeling procedures (e.g., fluorescent or magnetic) as well as high operating costs (reagents and consumables). Furthermore, the purification of target cells with little-to-no predetermined molecular biomarkers remains challenging. Recent biophysical studies have shown that alterations in the intrinsic biophysical properties of cells, such as size or deformability, could reflect changes in cellular phenotype and differences in cell lineage [8]–[13]. Isolation of precise cell populations using intrinsic biophysical properties would eliminate the need for potentially cell-damaging immunolabeling procedures. Such an approach would allow for cost-effective cell separation for therapies or downstream molecular biology-based assays.

Recent advances in microfluidic technologies have resulted in the development of numerous approaches, allowing simple, fast, and label-free target cell separation [14]. Among the many biophysical biomarkers, cell size is the most commonly used intrinsic cell property adopted for label-free microfluidic techniques, and many microfluidic devices have successfully demonstrated various potential applications [14]. Despite significant progress in technology development, however, most microfluidic cell separation systems have only demonstrated proof-of-concept separations with test biological samples, such as spiked cell samples or cell lines cultured *in vitro*. Here, to test the ability to collect cells from real tissue samples, we purified primary tissue digests containing adrenal cortical progenitor cells using a label-free microfluidic approach.

The adrenal cortex is located at the perimeter of the adrenal gland, a major hormone-secreting organ responsible for synthesis of steroid hormones [15], [16]. The condition where the cortex fails to generate essential hormones is called adrenal insufficiency (AI). AI can be caused by various human diseases (e.g., autoimmune disease and Addison's disease [17]) and result in an adrenal crisis, which leads to death if left untreated [15]. The current AI treatment requires lifelong daily replacement of hormones [17]. However, current therapies are less than ideal due to the considerable variation in the physiological demand for steroid hormones, which depends on time of the day and stress levels [17], [18]. Adrenal cortical tissue regeneration using stem cells from the adrenal cortex has been proposed to be a promising alternative [19]–[23]. Growing evidence has shown that there is a pool of cells in the adrenal cortex, which are potentially capable of continuous and lifelong regeneration of the tissue [18]–[23]. Progenitor cells, purified from either a patient's own or a healthy donor's biopsy, can be expanded *in vitro*, and these adrenal cells can be transplanted into the patient and potentially restore adrenal functionality or reverse adrenal insufficiency [24]. The purification of such cells, however, has been challenging because only a handful of intracellular molecular markers are available for the identification of adrenal cortical progenitor cells [16], [18], [23].

Previously, adrenal cortical progenitor cells were isolated based on increased intracellular cholesterol content within fully differentiated cells compared with progenitor cells, with differences potentially originating from different steroid hormone synthesis capabilities [25]. The intracellular cholesterol contents were examined by monitoring the fluorescent intensity of individual cells stained with Nile Red, a common dye that associates with cholesterol. After isolating cells using a conventional fluorescence-activated cell sorter (FACS), the previous study reported that the cells with lower Nile Red intensity (NR^dim^) expressed the steroidogenic factor 1 (Sf1) gene (i.e., the adrenal cortex gene) but lacked expression of zonal-specific adrenal cortical genes, such as Cyp11b1 (zona fasciculata) and Cyp11b2 (zona glomerulosa). This result suggests the successful enrichment and identification of a potential biomarker for a progenitor cell population.

However, the discrimination of progenitor cells from heterogeneous tissue digests was based on the intensity level of fluorescent stains, which can vary widely due to staining procedures and other challenges. For instance, dyes are often unsuitable for cells that will eventually be re-implanted in humans. Moreover, the function and proliferation rate of target cells isolated using immunoaffinity-based techniques could be adversely affected due to the strong binding between the labeling probes and targeting antigens [26], [27]. In contrast, label-free target cell purification techniques are generally less invasive and have lower operating costs than those based on current molecular-based biomarkers, providing a practical means to rapidly translate stem cell biology to regenerative medicine.

Here, we hypothesized that differences in intracellular cholesterol contents and phenotypic characteristics found in fully differentiated vs. progenitor cells would result in a variation in biophysical properties of such cells. It is known that cell adhesion molecules and junctional proteins are differentially expressed on cells with varying degrees of differentiation and lineage commitment [28]–[32]. Similar expression of adhesion molecules on differentiated cells will cause these cells to adhere more tightly to one another [33]. Additionally, fully differentiated somatic adrenal cortical cells would exhibit much stronger adhesion strength than less-differentiated progenitor cells because the higher cholesterol content of somatic cells may promote lipid clustering and enhance intercellular adhesive complexes. Thus, under the same condition of tissue digestion, differentiated cells would remain in multi-cellular clusters (e.g., larger apparent diameters), whereas the less-differentiated progenitor cells [32] could be dissociated as single cells. Recently, we have shown that cells of interest can be separated based on single-cell mechanical properties, such as size and deformability, utilizing only intrinsic hydrodynamic lift forces [34]. In brief, cells with different sizes or deformability experience different magnitudes of inertial lift forces, which induce lateral migration of flowing cells across streamlines and position cells at different locations corresponding to their mechanical properties. Within straight rectangular microchannels, larger and/or more deformable cells are positioned closer to the channel centerline, whereas smaller and/or stiffer cells are entrained near to the channel walls [34]. Similarly, the difference in mechanical properties associated with differentiation of adrenal cortical cells (i.e., variation in intracellular cholesterol content [25] or cell clustering from differential expression of cell-cell adhesion proteins [32]) should result in distinct lateral equilibrium positions in microscale inertial flow. In this manuscript, we present our results for the label-free isolation of murine adrenal cortical progenitor cells from adrenal gland digests using the deformability-activated cell sorter (DACS) [34].

## Results and Discussion

**Figure 1:**
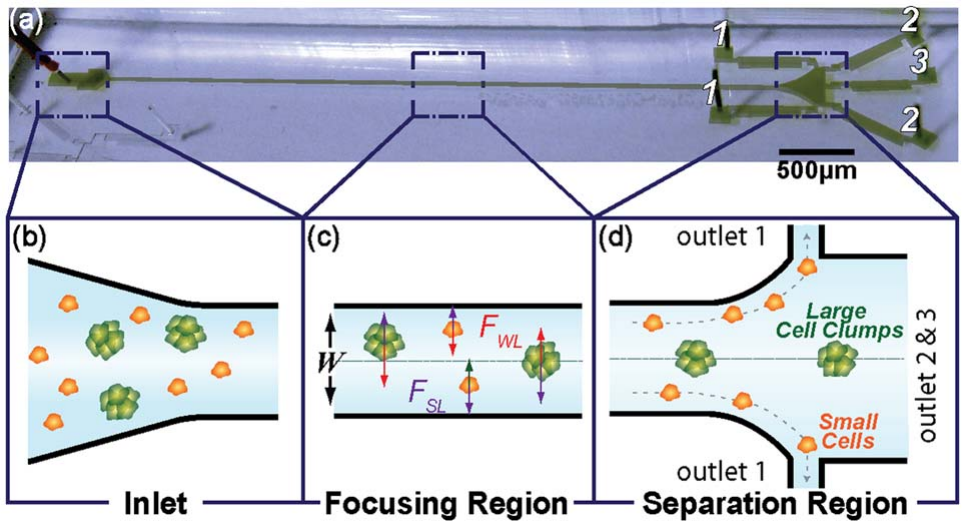
Label-free isolation of adrenal cortical progenitor cells. (a) The photograph of the microfluidic device used for progenitor cell isolation and (b–d) schematic showing the inertial focusing of living cell clumps in microscale flow. (b) Solution containing randomly distributed heterogeneous tissue digest is injected at the inlet of the device and (c) flowing cells experience two lateral forces, namely wall effect lift, F_WL_, and shear-gradient lift force, F_SL_, as they travel through the straight focusing region, and these forces induce lateral migration of cells and focus them at different locations base on the size (i.e., larger cells were closer to the channel center, and smaller cells were closer to the channel walls). (d) Differentially focused flowing cells can be directed and collected at designated outlets based on apparent-diameter variation. All schematics represent the top view of the microfluidic device.

**Figure 2:**
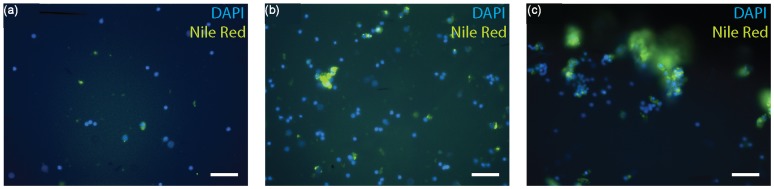
Label-free separation of murine adrenal cortical progenitor cells using deformability activated cell sorting technique. Fluorescent images of cells collected at (a) outlet 1, (b) outlet 2, and (c) outlet 3. Cells were stained post-collection with DAPI (blue) and Nile Red (green) to identify intracellular lipid droplets and nuclei, respectively. There was a significantly lower number of cells expressing high levels of lipid droplet contents (Nile Red bright, somatic adrenal cortical cells) found in the fraction collected from outlet 1, and the population of Nile Red bright cells was the highest in the fraction collected from outlet 3. Scale bar is 50 µm.

Murine adrenal cortical progenitor cells were purified from a heterogeneous tissue digest using DACS in a simple and label-free manner. Heterogeneity in biophysical properties of digested cells in the injected solution allows the inertial focusing mechanism to locate subpopulations differentially at distinctive distances from the channel walls (i.e., lateral equilibrium position). Based on these differences in lateral equilibrium position, smaller single cells were directed toward the outermost outlets (outlet 1 in Figure 1(a)) and purified from the larger cells and cell clusters in the tissue digest solution. Fluorescence imaging after separation with DACS (Figure 2) showed that cells with little or no cholesterol content (i.e., dim Nile Red intensity) were collected at outlet 1, whereas other cell types with higher content (i.e., bright Nile Red) were enriched at outlet 3. In addition, cells with higher cholesterol content were collected in the form of multicellular clusters (Figure 2(c)), whereas cells collected in the form of single cells were shown to have lower levels of cholesterol (Figure 2(a)). This result indicates that the more differentiated adrenal cells, i.e., containing higher content of cholesterol, were collected in the form of multicellular clusters at the center outlets, whereas the progenitor cells were collected in the form of single cells at the outermost outlets. This could be attributed to different phenotypic characteristics that cells with varying degrees of differentiation exhibit [28]–[32], as we had hypothesized. Multicellular differentiated cell clusters with tighter adhesive strength will have much larger apparent diameters than their actual sizes as they tumble through the focusing region (see Figure 1 (c)), as we had previously found for non-spherical particles [35], suggesting that the large size difference between clustered differentiated cells and individually dispersing progenitor cells enabled the label-free purification of progenitor cells used the current device.

**Figure 3:**
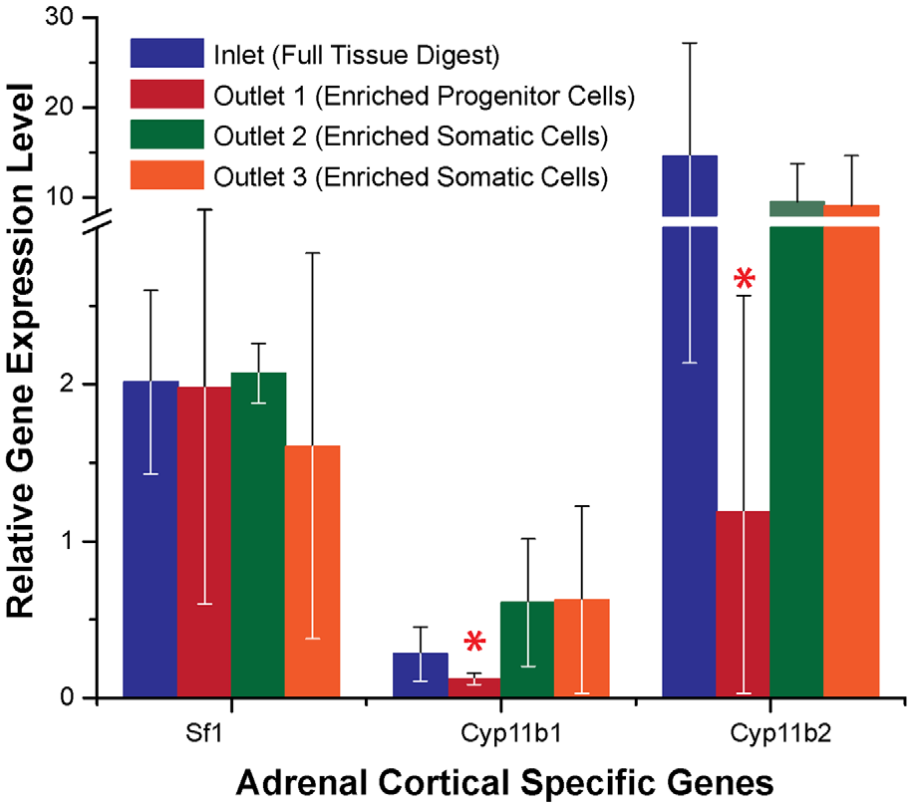
Relative gene expression levels of Sf1, Cyp11b1, and Cyp11b2 of all adrenal cells collected at each fraction of outlets. Each adrenal specific gene expression level was normalized to that of pooled neonatal adrenal glands. The levels of Sf1 were similar (P = 0.9) for all conditions, whereas Cyp11b1 and Cyp11b2 were differentially expressed for fractions collected at different outlets. The expression of zonal-specific genes (Cyp11b1 and Cyp11b2) was significantly lower in the fraction of outlet 1 than that for the rest of samples. Red asterisks indicates P<0.05 compared with NR bright cells from outlet 3. Error bar represents the standard deviation of measurements from two animals.

Gene expression measurements further confirmed that the outermost outlet 1 and the center outlet 3 contain different cell populations (see Figure 3). The tissue-specific gene, Sf1, was expressed with relatively similar level compared with both the control (not flowed through the device) and experimental groups (p = 0.9). However, the expression of zonal-specific genes, Cyp11b1 and Cyp11b2, were substantially suppressed for the cells collected from the outermost outlets, indicating that those cells were least differentiated (P<0.05). Collectively, the results show that more differentiated multicellular clusters of adrenal cells, expressing higher levels of zonal-specific genes (Cyp11b1 and Cyp11b2) were enriched at inner outlets (outlet 2 and 3), while the expression levels of those genes were significantly lower for the adrenal cortical progenitor cells collected at the outermost outlets (outlet 1) [25]. The clear differences in gene expression profiles of cells collected at different outlets suggest that differences in differentiation of adrenal cortical cells can be reflected in biophysical property dissimilarities (e.g., adhesive properties and relative tendency to remain in clusters after tissue dissociation). Thus, label-free adrenal progenitor cell purification based solely on biophysical properties appears to be feasible.

**Figure 4:**
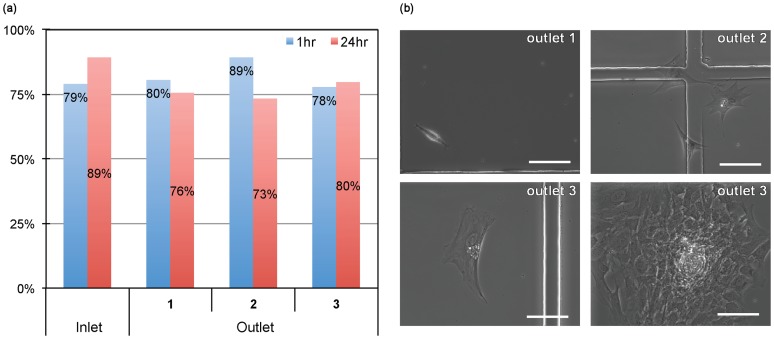
Processed cells remain highly viable. (a) Colorimetric viability test showed that cells flowed through the device remain highly viable similar to control cells, which have not been processed. (b) Collective bright field images of 10 day cultured cells. Various cell types with different morphologies were observed. Scale bar is 100 µm.

In addition, viability tests and 10-day *in vitro* culture results showed that those processed primary cells were not adversely affected by the inertial flow. The viability of the processed cells was not significantly lower than that of the control samples, and more than 70% of the cells remained viable 24 hours post-processing (see Figure 4 (a)). Furthermore, we were able to culture the collected samples for 10 days *in vitro* by following previously developed protocols [36]. Interestingly, various cell types with distinct morphology were observed during the course of the 10-day culture. Only cells collected from the center outlets proliferated and resided in clusters, consistent with previous histological observation [37]. Despite the fact that further study will be needed to identify definitive phenotypes of subpopulations collected from each outlet, the current study suggests that the purification of target cells from the tissue digest using DACS would enable further downstream analyses to identify specific culture conditions or new molecular/genetic biomarkers for the collected subpopulations.

Utilizing the substantial difference in lateral equilibrium positions between single cells and clusters from heterogeneous tissue digests, viable adrenal cortical progenitor cells were purified in a simple, passive, and label-free manner. The throughput of the current process was estimated to be 24,000 cells/min when the device operated at 60 µL/min; approximately 2 million cells were harvested from one mouse (2 adrenal glands) and diluted in 5 mL Knockout (KO) media prior to separation. Our previous study has demonstrated that the current flow condition approaches the maximum flow rate and throughput of a single DACS, given that a further increase in fluid inertia would result in uniform focusing positions regardless of cell size variation [38]. Although the current throughput is slower than conventional FACS systems, the approach does not require labeling and a 45-fold improvement in throughput is anticipated using previously demonstrated parallel geometries for the device [39]. Moreover, a higher level of purity for collected progenitor cells can be achieved by cascading the devices in series [40]. Furthermore, it is important to note that the current system is very robust, autonomously and stably operating for >3 hours while maintaining uniform flow at all outlets. Cell death is reduced by including low-shear transition designs, minimizing cell rupture and cross-contamination. Finally, we previously confirmed that there were no significant changes in gene expression for cells pre- and post-processing with the system [34].

## Materials and Methods

### Device Design

To isolate adrenal cortical progenitor cells from the digested tissue samples, we used the DACS as previously reported [34]. Briefly, the device is composed of a straight focusing channel (*W* = 40 µm, *H* = 90 µm and *L* = 4.5 cm), a gradually expanding region, and 5 branched outlets with fluidic resistors. The angle between the channel wall and the flow direction in a gradual expansion was increased 2° for every 100 µm. The dimension of a gradual expansion was carefully designed through numerous empirical tests to create a smooth and low transition in shear that processed cells would experience. The low-shear transition design was essential to confine focused cells to their predetermined streamlines while enhancing *X_eq_* differences among cell types, as well as maintaining minimum distances between cells flowing toward designated outlets, without sacrificing the purity of collected samples. Additionally, long serpentine channels with dominating fluidic resistance were incorporated into outlet designs to ensure undistorted and uniform flow through individual outlets during the entire course of the purification process regardless of small fluctuations in fluidic resistances. Regardless, those resistors were designed to have negligible shear effects on flowing cells (e.g., 60% shorter in length, 30% wider width, and 1/5 of the volumetric flow rate compared with the focusing region). Detailed dimensions and empirical supporting evidence can be found elsewhere [34]. The conventional soft lithography technique using PDMS was used for device fabrication. In brief, a negative photoresist (KMPR 1050, Microchem) was spin-coated onto a 4″ silicon wafer followed by replica molding of PDMS (Sylgard 184, Dow Corning). After the interconnections were created using a pin vise (Pin vise set A, Technical Innovations, LLC), the casted device was enclosed by permanently bonding it to a slide glass using air plasma (Plasma Cleaner, Harrick Plasma).

### Reagents and Media

Dulbecco's modified Eagle's medium and Ham's F12 medium (DMEM/F12), KnockOut™ DMEM, Hank's balanced salt solution (HBSS), fetal bovine serum, horse serum, KnockOut™ serum replacement, GlutaMAX™, and antibiotics were purchased from Invitrogen (Carlsbad, CA). Collagenase I, deoxyribonuclease I, and bovine serum albumin were purchased from Sigma-Aldrich (St. Louis, MO). Collagen sponge Helistat was purchased from Integra LifeSciences (Plainsboro, NY). Adrenal Media was prepared with DMEM/F12 medium, 15% horse serum, 2.5% fetal bovine serum, and antibiotics. Knockout (KO) media was prepared with KnockOut™ DMEM, 15% KnockOut™ serum replacement, GlutaMAX™, and antibiotics.

### Animals

Female C57/BL6 mice, 8 weeks in age (20–22 g), were purchased from Charles River Laboratory (Wilmington, MA). All animals were maintained in an animal barrier as a non-breeding colony in a temperature- and light-controlled room and were allowed free access to food and water. In each experiment, C57 mice served as donors. The use of the animals was approved by the UCLA Animal Research Committee (IRB 2003-178).

### Isolation of Adrenal Cells from Adrenal Glands

Adrenal glands were removed from mice after euthanasia. After removing the surrounding fat, the harvested adrenal glands were incubated in the digestion mixture at 37°C for 1 hour with gentle shaking. The digestion mixture consisted of 10 mL of HBSS containing 2 mg/mL collagenase I, 0.05 mg/mL DNase I, and 5 mg/mL bovine serum albumin. After dispersing the cells through a pipette, the cells were washed with Adrenal Media and then filtered through a 70-µm strainer (Millipore, Bedford, MA). Cells collected from two adrenal glands were suspended with KO media (5 mL).

### Label-free Enrichment of Adrenal Cortical Progenitor Cells

5 mL of cell suspensions containing 400,000 cells/mL were loaded into a glass syringe (Hamilton GASTIGHT®), and the solutions were injected into DACS while being continuously agitated using a custom-built cell shaker. The overall flow rate was maintained at 60 µL/min using a syringe pump (Harvard Apparatus PHD2000), as we had previously found that the maximum separation was achievable at this flow condition using the current device [34]. The system was sufficiently robust to run more than 3 hours without a change in flow pattern due to clogging. The fractions collected from outlet 1 (i.e., progenitor cell collection outlet), outlet 2, and outlet 3 (i.e., somatic cell collection outlet) were labeled accordingly for the downstream analysis to determine gene expression by qRT-PCR. Sorted adrenal cells were stained with Nile red (1 mg/mL in Dimethyl Sulfoxide) and DAPI (3 µM in Phosphate buffered saline) by adding to the cell suspensions at 1∶100 and 1∶10 dilutions, respectively, in order to examine their lipid contents and nuclei using fluorescence microscopy (Eclipse Ti, Nikon, Japan). Portions of processed samples from each outlet group were saved and used for cell viability tests.

### RNA Extraction and qRT-PCR

RNA extraction from sorted and unsorted cells was performed with RNeasy Mini kit (Qiagen Valencia, CA), following the manufacturer's protocol. The expression of specific mRNAs was obtained with quantitative reverse transcriptase polymerase chain reaction (qRT-PCR), Qiagen qRT-PCR kit. Primers and probes for amplifying adrenal gland genes of interest (Sf1, Cyp11b1 and Cyp11b2) were purchased from Operon Technologies (Valencia, CA), while the housekeeping gene (GAPDH) primers were purchased from Applied Biosystems (Foster City, CA). qRT-PCR data for each sample were obtained using ABI PRISM 7900 Sequence Detection System. The reverse transcription was performed at 50°C for 30 min, followed by the initial activation at 95°C for 15 min. Then, samples were processed for 45 cycles of 94°C for 15 sec and 60°C for 60 sec. The threshold cycle number for each sample indicates the cycle number at which the fluorescence signal exceeded the preset threshold value, which was the value ten times greater than the standard deviation of the baseline. Gene expression results were normalized to that of the mRNA level in pooled neonatal adrenal glands using the comparative C_T_ method [41], 0.5^(C^_T_^−C^_A_^)−(C^_G_^−C^_B_^)^. Here, C_T_ and C_G_ are the threshold cycle number for adrenal cortical gene and GAPDH in the sample (e.g., inlet and outlet 1∼3), respectively, whereas C_A_ and C_B_ are the cycle number for the same adrenal cortical gene and GAPDH in the pooled neonatal adrenal glands, respectively. RNA from multiple adrenal glands of neonatal mice was extracted and pooled in order to prepare a large batch of adrenal RNA. It served as a constant normalization standard for multiple qRT-PCR analyses. Statistically significant differences between the two sets of mRNA levels were determined using Student's t-test.

### Cell Viability and Long-term Culture

To assess whether the current enrichment method adversely affected processed cells, we performed colorimetric live/dead assay using Calcein AM and Ethidium homodimer-2 (Invitrogen) in addition to long-term culture with KO media following previously established protocols [36]. For the colorimetric live/dead assay, the ratio between the number of live and dead cells was determined by counting the stained cells in fluorescent images taken from one entire individual well of a standard 96-well plate (132 stitched images). For long-term cultures, the processed cells were seeded in μ-dish with grids (ibidi GmbH, Germany) and cultured for 10 days. The media was replaced every 48 hours and followed by monitoring of cell proliferation.

## Conclusions

We have demonstrated that target cells in the form of dispersed single cells can be passively purified from multicellular clusters from complex heterogeneous tissue digests using DACS. Putative adrenal cortical progenitor cells with little-to-no cholesterol content can be isolated as single cells at the outermost outlets, whereas more differentiated adrenal cells with higher cholesterol content can be collected as multicellular clusters at the inner outlets. Our preliminary results show that DACS has practical applications in tissue engineering and regenerative medicine, which often require the processing of fragile primary cells or stem cells with significant yields/throughputs. Furthermore, the ability to isolate multicellular clusters from heterogeneous cell solutions can provide a useful tool to study native organized structures, which is often required for cancer prognostics [42] and/or immunophenotypical, physiological, and intact morphological studies for a tissue-level understanding of biology [43]–[46].
